# Timing of adverse events among voluntary medical male circumcision clients: Implications from routine service delivery in Zimbabwe

**DOI:** 10.1371/journal.pone.0203292

**Published:** 2018-09-07

**Authors:** Caryl Feldacker, Aaron F. Bochner, Vernon Murenje, Batsirai Makunike-Chikwinya, Marrianne Holec, Sinokuthemba Xaba, Shirish Balachandra, John Mandisarisa, Vuyelwa Sidile-Chitimbire, Scott Barnhart, Mufuta Tshimanga

**Affiliations:** 1 International Training and Education Center for Health (I-TECH), Seattle, Washington, United States of America; 2 Department of Global Health, University of Washington, Seattle, Washington, United States of America; 3 Department of Epidemiology, University of Washington, Seattle, Washington, United States of America; 4 International Training and Education Center for Health (I-TECH), Harare, Zimbabwe; 5 Ministry of Health and Child Care, Harare, Zimbabwe; 6 U.S. Centers for Disease Control and Prevention, Harare, Zimbabwe; 7 Zimbabwe Association of Church-related Hospitals (ZACH), Harare, Zimbabwe; 8 Department of Medicine, University of Washington, Seattle, Washington, United States of America; 9 Community Health Intervention Project (ZiCHIRe), Harare, Zimbabwe; University of Illinois at Chicago, UNITED STATES

## Abstract

**Background:**

Timing of routine follow-up visits after adult male circumcision (MC) differs by country and method. Most men do not attend all routine follow-up visits. This cross-sectional study aimed to further understanding of AE timing within a large-scale, routine, MC program to improve patient safety.

**Methods:**

From 2013–2017, ZAZIC consortium performed 192,575 MCs in Zimbabwe; the reported adverse event (AE) rate was 0.3%. Three scheduled, routine, follow-up visits intend to identify AEs. For surgical MC, visits were days 2, 7 and 42 post-procedure. For PrePex (device-based), visits were days 7, 14 and 49. Descriptive statistics explored characteristics of those patients with AEs. For each MC method, chi-square tests were used to evaluate associations between AE timing (days from MC to AE diagnosis) and factors of interest (age, AE type, severity).

**Results:**

Of 421 AEs, 290 (69%) were surgical clients: 55 (19%) AEs were ≤2 days post-MC; 169 (58%) between 3–7 days; 47 (16%) between days 8–14; and 19 (7%) were ≥15 post-MC. Among surgical clients, bleeding was most common AE on/before Day 2 while infections predominated in other follow-up periods (p<0.001). Younger surgical MC patients with AEs experienced AEs later than older clients (p<0.001). Among 131 (31%) PrePex clients with AEs, 46 (35%) were ≤2 days post-MC; 59 (45%) between 3–7 days; 16 (12%) between days 8–14; and 10 (7%) ≥15 post-MC. For PrePex clients, device displacements were more likely to occur early while late AEs were most commonly infections (p<0.001).

**Conclusion:**

Almost 23% of surgical and 8% of PrePex AEs occurred after Visit 2. Later AEs were likely infections. Clinicians, clients, and caregivers should be more effectively counseled that complications may arise after initial visits. Messages emphasizing attention to wound care until complete healing could help ensure client safety. Younger boys, ages 10–14, and their caregivers would benefit from improved, targeted, post-operative counseling.

## Background

As voluntary medical male circumcision (MC) expands in sub-Saharan Africa, consideration of program quality should match efforts to increase program productivity. One way to ensure patient safety is through appropriate patient follow-up to identify and treat adverse events. Adverse events (AE) for MC procedures are uncommon: an AE rate of 2% is regarded as the global standard of MC safety and the adopted standard of care for Zimbabwe [1, 2]. Also, few reports identify men with AEs resulting in permanent disability or death. MC expansion reached almost 14.5 million MCs by the end of 2015 [3], and global MC programs aim to reach 5 million men, annually, through 2021[4]. This MC progress and scale up suggests that, annually, tens of thousands of men are expected to experience an AE.

The timing of routine follow-up visits in MC programs is designed to ensure patient safety by identifying, treating and managing complications. Timing differs between surgical and device-based (PrePex) MC: PrePex MC requires a device removal visit approximately seven days post-placement and healing is slower than for surgical MC [5, 6]. Although routine follow-up timing may differ by country, in Zimbabwe where MCs are done surgically or using PrePex, three follow-up visits are scheduled to ensure quality service provision and patient care: *Visit 1* (Day 2 for surgical; Day 7 for PrePex for device removal); *Visit 2* (Day 7 for surgical and Day 14 for PrePex); and, *Visit 3* (Day 42 for surgical and Day 49 for PrePex) [7]. These visits intend to identify early AEs, verify appropriate healing, and identify any adverse events for immediate treatment and management. In other countries in sub-Saharan Africa, review visits adhere to a minimum review schedule emphasizing follow-up visits 1 and 2 [8] or at least one review within 14 days of procedure [9, 10]. Most men do not attend all scheduled post-operative visits [11].

**Fig 1:**
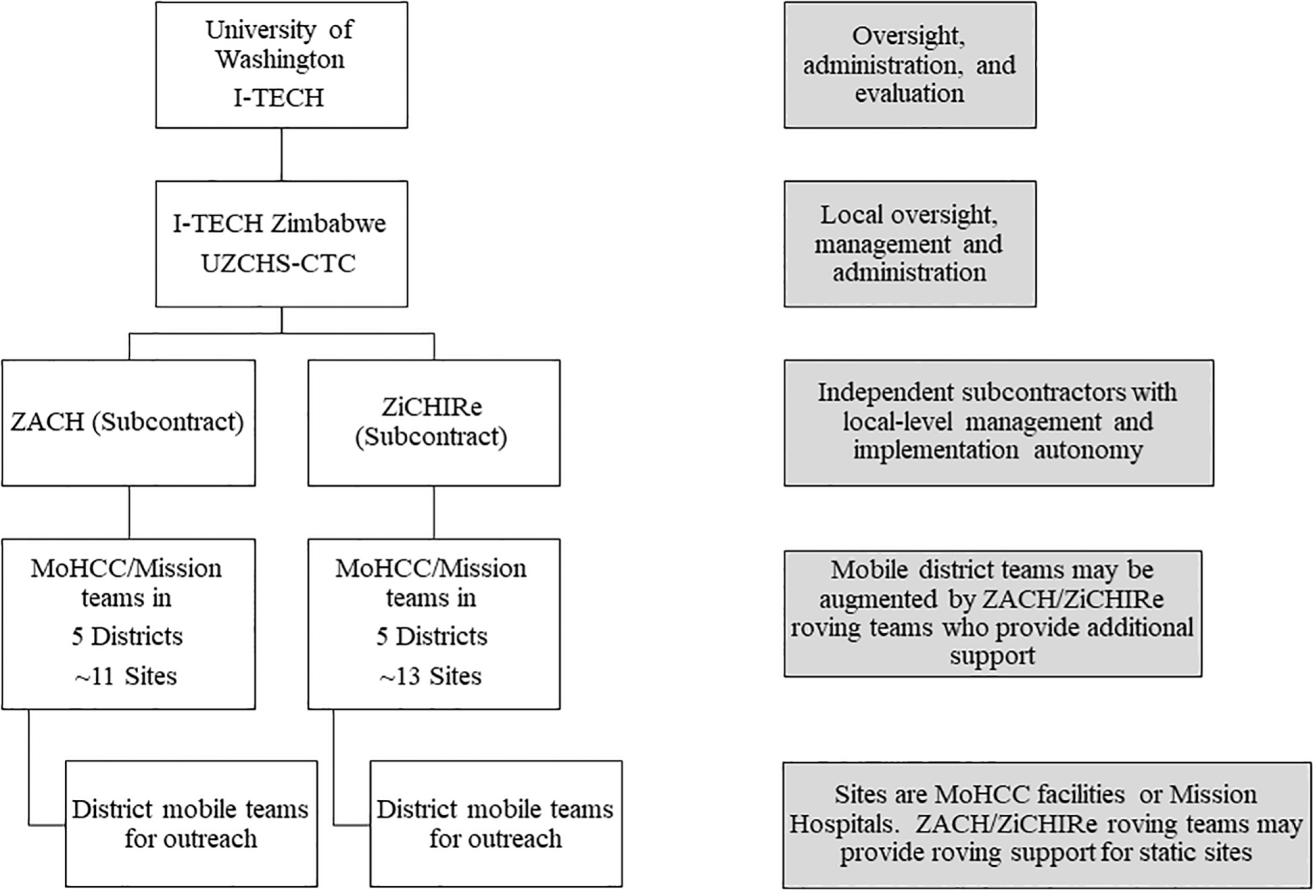
ZAZIC Organizational Model. ZiCHIRe: Zimbabwe Community Health Intervention Project; ZACH: Zimbabwe Association of Church-related Hospitals; UZCHS-CTC: University of Zimbabwe College of Health Sciences—Clinical Trials Center.

In Zimbabwe, the ZAZIC consortium (an acronym created from partners’ names) comprised of three, local implementing partners began implementation of MC in coordination with the Ministry of Health and Child Care (MoHCC) in 2013 using an integrated program model in 21 (20 rural and one urban) districts (Fig 1). Within the ZAZIC program, men are instructed to come in at any time, before or after scheduled visits, if they are concerned about an AE. Most men attended Visits 1 and 2; however, fewer than 30% attended Visit 3 to verify complete healing (Marongwe, personal communication, 2016). MoHCC MC guidelines include active tracing for clients who do not return for Visits 1 and 2; there is no patient tracing for Visit 3. However, although no patients are actively followed after Visit 2, AEs may be detected after this time as demonstrated previously [12–14].

In this cross sectional analysis, we reviewed all documented moderate and severe adverse events reported within the ZAZIC consortium MC program. Through the end of March 2017, the program performed 192,575 procedures. Previously, we presented findings on moderate and severe AE rates for both surgical and PrePex MCs from one year of MC program implementation (October 2014-September 2015). We found that AEs, overall, were uncommon: 0.3% (116/41,416) of surgical and 1.2% (40/3,452) of PrePex clients experienced a moderate or severe AE. Results also indicated that PrePex clients were more likely to experience an AE and that younger patients ages 10–14 were more than three times more likely to experience infection as compared to their older peers, ages 20 and older [15].

In this paper, we complemented that analysis by describing AEs from routine program implementation from March 2014-March 2017. Using an aggregate database of AEs, we focused on time from procedure to AE diagnosis and explored factors that may have influenced the timing of AEs by MC method (surgical or PrePex). Further understanding of the timing of AEs may help inform patient counseling and improve program quality in Zimbabwe. These findings may also help improve MC program implementation and patient safety in the region, overall.

## Methods

### Study population

Between March 2013 and March, 2017, ZAZIC supported MC services for males age 10 and older, performing 192,575 MCs within 21 districts: 20,987 PrePex (10.9%) and 171,588 surgical (89.1%)[16]. Three circumcision techniques were used over the study period: surgical forceps-guided MC was approved for clients ages 15 and above; surgical dorsal slit was approved for clients ages 10 and above; and PrePex was approved for clients ages 15 and above. Before July 2016, PrePex was only approved for men aged 18 and older. Clients over age 15 chose between surgical and PrePex; clinicians chose the specific surgical method.

Overall, the average monthly AE rate for both moderate and severe AEs was 0.3% with a range of 0% to 2.3% as reported in ZAZIC monthly routine monitoring data [15]. Over the implementation period, approximately 40% of all ZAZIC MCs were performed among boys ages 10–14, 25% were among clients ages 15–19, and 35% were ages 20 and above.

### Data collection

From 2013, a MoHCC Monthly Return Form of site-based summary statistics (including # of MCs; client age groups; # of AEs) was completed by sites at the end of each month, reporting the number of moderate or severe AEs to MoHCC and ZAZIC. In July 2014, the President’s Emergency Plan for AIDS Relief (PEPFAR) began tracking notifiable (moderate or severe) AEs [17]. In accordance with this PEPFAR policy, from July 2014, ZAZIC added additional details on each moderate and severe AE (client age, MC date, AE date, AE type, AE severity) to the ZAZIC-specific monthly program report. ZAZIC also rolled out an Adverse Event Review Tool (AE Review Tool), a ZAZIC-developed form that collected detailed AE history, examination findings, management, and clinical outcomes for each notifiable AE. Site clinicians completed the AE Review Tool first. ZAZIC clinicians then reviewed the identification, treatment, and management for quality assurance. Inconsistencies in the AE Review Tool and additional information were gathered by phone calls or site visits. Adverse events were categorized according to standard PEPFAR definitions [10]. By October 2014, all ZAZIC sites completed the AE Review Tool with fidelity for each AE. For this study, AE data from ZAZIC-specific monthly program reports was aggregated into a database without individual identifiers. Supplemental information was added from the AE Review Tool, when needed, to complete the study database. Among clients who experienced more than one category of AE, only the most severe AE was included in the analysis.

### Definitions

In general, severe AEs were those that required surgical intervention or hospitalization due to other than logistical reasons. Any AE not classified as severe, but which required intervention by a health care provider or medication, was considered moderate. Mild AEs, those that required mostly reassurance, were not reported within the ZAZIC consortium nor included in this analysis. Follow-up visits 1, 2, and 3 conformed to Zimbabwe MoHCC guidelines [7]. Age was categorized into PEPFAR reporting groups: ages 10–14; ages 15–19; and ages 20 and older. For both methods, timing of AE was determined using the number of days between surgery or PrePex placement (Day 0) and the day the client was diagnosed with the AE at the clinic. Timing of AEs was categorized for this study as occurring ≤ Day 2; Days 3–7; Days 8–14; and Day 15+.

### Data analysis

Summary statistics detailed demographics of clients with AEs, presented by surgical and PrePex. Chi-square tests explored significant associations between timing of AEs and individual characteristics by MC method (surgical and PrePex) as eligibility and follow-up procedures differed. Day zero was the day of surgical circumcision or PrePex placement. This analysis was conducted using STATA 12 [18].

### Ethics

The information included in this cross-sectional analysis were routinely-collected, ZAZIC programmatic data. Data were entered without individual identifiers, including patient name and unique program ID, into an aggregate database used exclusively for program monitoring. The study received a Non-Research Determination from the University of Washington.

## Results

### Demographics and AE characteristics by MC method

Overall, 617 moderate and severe AEs were reported between March 2013 and March 2017. Of those, 421/617 (68%) AEs had complete information on the AE Review Tool and were included in further analysis (data in S1 Text). Of the 421 AEs, 179 (42.5%) were among boys ages 10–14; 122 (29.0%) were boys ages 15–19; and 120 (28.5%) were among those ages 20+. Of those same 421 AEs, 257 (61%) were moderate and 164 (39%) were severe. Surgical MCs accounted for 290 (68.9%) and PrePex clients contributed 131 (31.1%) of all included AEs. Among the 421 AEs, 13 (3%) were reported within the intra-operative period: 3 PrePex and 10 surgical. These were included as having occurred on Day 0.

As MC eligibility and follow-up procedures differ for surgical and PrePex clients, client and AE characteristics were dissimilar (Table 1). Among the 290 surgical AEs, median age was 13 years (IQR: Inter-quartile range (11; 16). Median time to AE was 7 days (IQR: 4; 7): the majority of AEs (169 (58.3%)) occurred between 3 and 7 days, but 19 clients (6.6%) had an AE 15 or more days post MC. The majority of surgical AEs were moderate (227 (78.3%)). One moderate AE was reported on day 178 because the client had been traveling for several months post MC. Of the 63 (21.7%) severe AEs, 31 (49%) were among boys ages 10–14.

**Table 1:** Characteristics of clients with AEs by MC type: Surgical and PrePex.

	Surgical (n = 290) N (%)	PrePex (n = 131) N (%)	Total (n = 421) N (%)
Age			
Mean	15.0	25.3	18.2
Median (IQR)	13 (11; 16).	23 (19; 30)	16 (12;21)
Client age group			
10–14	179 (61.7)	-	179 (42.5)
15–19	77 (26.6)	45 (34.4)	122 (29.0)
20+	34 (11.7)	86 (65.6)	120 (28.5)
AE severity			
Moderate	227 (78.3)	30 (22.9)	257 (61.1)
Severe	63 (21.7)	101 (77.1)	164 (38.9)
Facility site type			
Static	100 (34.4)	60 (45.8)	160 (38.0)
Outreach	179 (61.7)	65 (49.6)	244 (58.0)
Unknown	11 (3.8)	6 (4.6)	17 (4.0)
MC type			
Dorsal slit	169 (58.3)	-	169 (40.1)
Forceps guided	94 (32.4)	-	94 (22.3)
Unspecified surgical	26 (8.9)	-	26 (6.2)
PrePex	-	131	131 (31.1)
Unknown	1 (0.03)	-	1 (0.2)
Days to AE			
Mean	7.45	5.55	6.8
Median (IQR)	7 (4; 7)	3 (2; 7)	7 (3; 7)
AE timing			
≤ Day 2	55 (19.0)	46 (35.1)	101 (24.0)
Days 3–7	169 (58.3)	59 (45.0)	228 (54.2)
Days 8–14	47 (16.2)	16 (12.2)	63 (15.0)
Day 15+	19 (6.6)	10 (7.6)	29 (6.9)
PEPFAR AE type			
Infection	209 (72.1)	23 (17.6)	232 (55.1)
Device displacement	-	94 (71.8)	94 (22.3)
Bleeding	59 (20.3)	5 (3.8)	64 (15.2)
Other (swelling, etc.)	10 (3.5)	3 (2.3)	13 (3.1)
Scarring/disfigurement	9 (3.1)	1 (0.8)	10 (2.4)
Pain	2 (0.7)	5 (3.8)	7 (1.7)
Anesthesia-related	1 (0.3)	-	1 (0.24)

IQR: Inter-quartile range (Q1; Q3).

Among the 131 PrePex AEs, median age was 23 years (IQR: 19; 30). Median time to AE was 3 days (IQR: 2; 7): the largest proportion of men (59 (45.0%)) reported AEs between days 3 and 7, but 10 clients (7.6%) had an AE 15 or more days post MC. The majority of AEs were severe (101 (77.1%)). Among the severe AEs, 68 (67%) were among men ages 20 and older. Among clients experiencing an AE who would have been age-eligible for either method (aged 18 or older), the mean age of surgical clients (n = 52) was 25.1 years; the mean age of PrePex clients (n = 124) was 25.9 years.

### Relationship between client age, AE type, and timing of AE, by MC method

**Fig 2:**
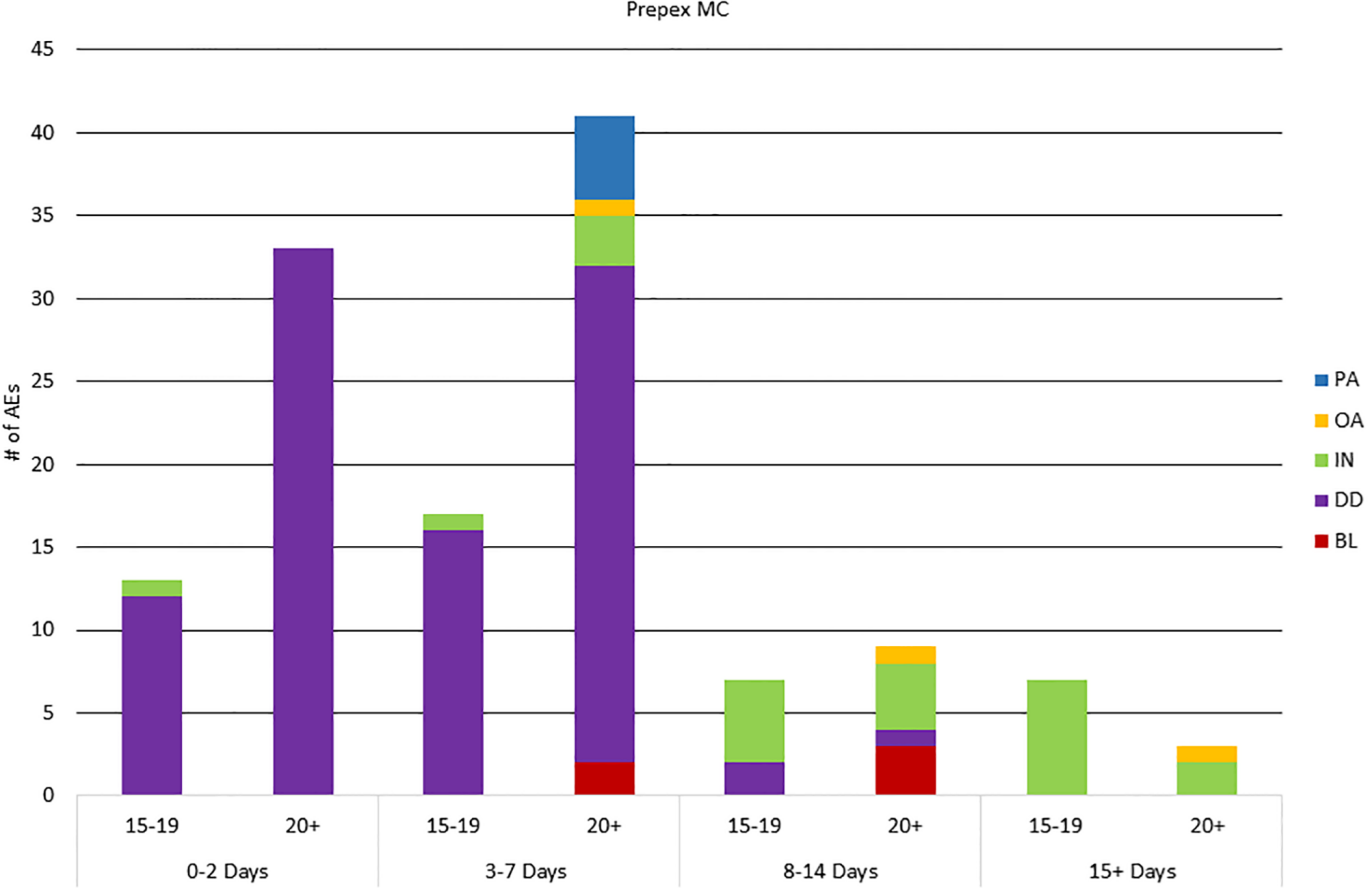
PrePex AEs by age group and AE timing. PA: pain; OA: other AE (e.g., swelling); IN: infection; DD: device displacement; AN: anesthesia-related; BL: bleeding; SD scarring/disfigurement.

**Fig 3:**
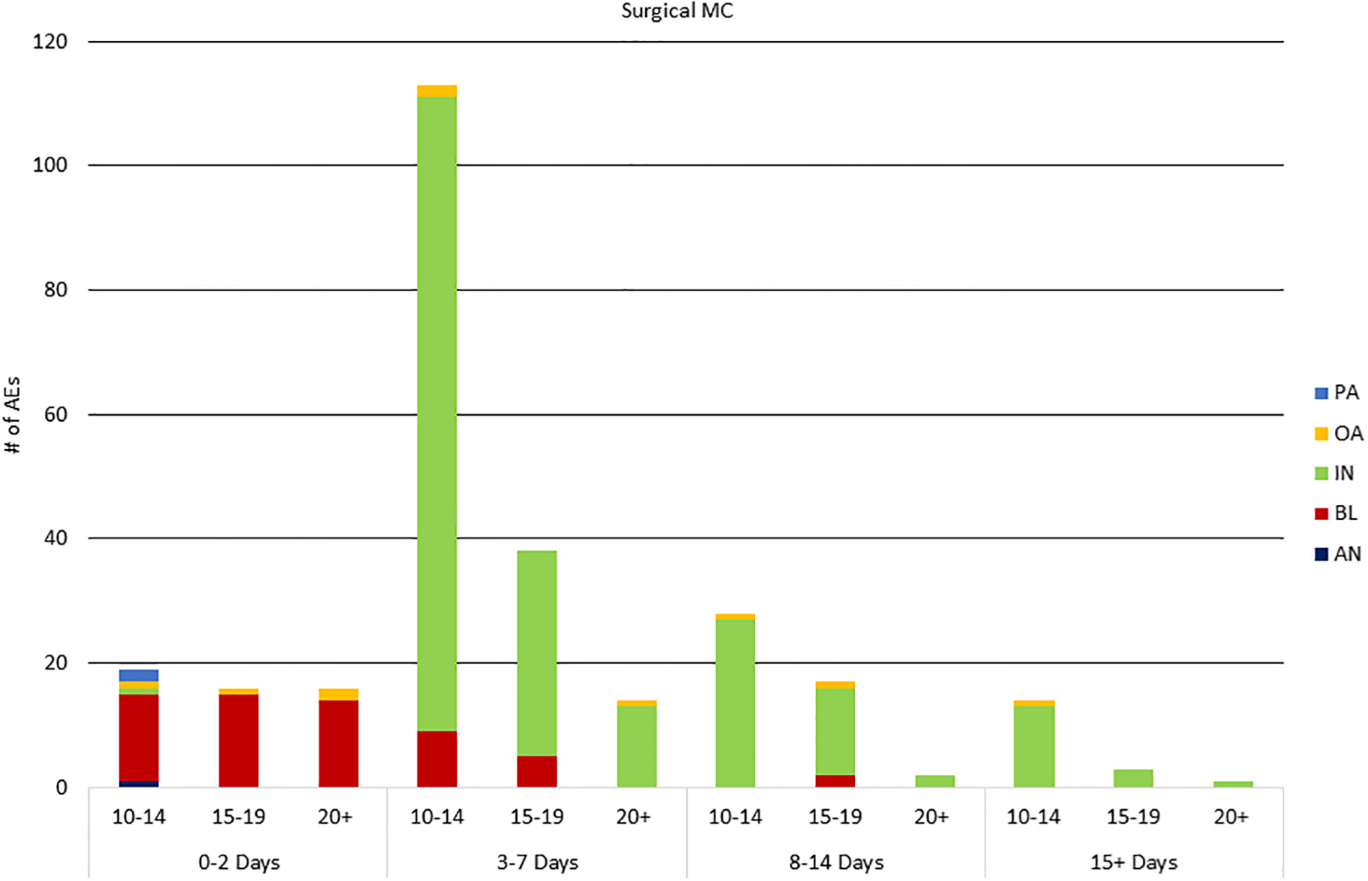
Surgical AEs by age group and AE timing. PA: pain; OA: other AE (e.g., swelling); IN: infection; DD: device displacement; AN: anesthesia-related; BL: bleeding; SD scarring/disfigurement.

Fig 2 (PrePex) and Fig 3 (surgical) illustrate differences in AE type over time, by method. For both surgical and PrePex, the types of AEs varied over the healing period. Among surgical AEs, bleeding was the most common AE among all age groups in days 0–2 which included Visit 1 on Day 2. Infection was most common in days 3–7, including scheduled Visit 2 on Day 7. After Visit 2, infection was the leading AE among all age groups. Only 3 surgical AEs occurred after Day 21: one infection on Day 24, one infection on Day 42, and one scarring/disfigurement on Day 178. Among PrePex clients with AEs, device displacements clearly dominated AEs identified during days 0–7, while the device was in place, including the scheduled PrePex removal on Visit 1 on Day 7. Infection was the most common AE in days 8–14, including scheduled Visit 2 on Day 14. After Visit 2, infection was most common in both age groups. Only 1 PrePex AE, an infection of Day 26, occurred after Day 21.

### Associations between AE characteristics and time to AE diagnosis

Several factors were associated with timing for both surgical and PrePex AEs (Table 2). Among surgical AEs, the timing of AEs differed significantly by age group (p<0.001). The proportion of AEs, by age, was similar on Days 0–2. However, younger boys (ages 10–14) comprised 79% (15/19) of the AEs that occurred on or after Day 15: younger boys appeared to have AEs diagnosed later than their older peers. AE severity also varied significantly over time (p = 0.004). Although moderate AEs predominated in all time periods, severe AEs comprised more than 30% of both early (on/before Day 2) and late (on/after Day 15) AEs. AE type also appeared to differ over time (p<0.001): of AEs occurring on or after Day 8, the vast majority were infections suggesting that this AE type presented later by comparison to other AE types.

**Table 2:** Results of associations* between age, severity, and AE type and timing of AEs, by MC method.

Row percentages	Surgical N (n = 290) %**					PrePex N (n = 131) %**				
	≤ Day 2	Days 3–7	Days 8–14	Days 15+	p-value	≤ Day 2	Days 3–7	Days 8–14	Days 15+	p-value
Client age group					<0.001					0.060
10–14	21 11.7%	115 64.3%	28 15.6%	15 8.4%		-	-	-	-	
15–19	17 22.1%	40 52.0%	17 22.1%	3 3.9%		13 28.9%	18 40.0%	7 15.6%	7 15.6%	
20+	17 50.0%	14 41.2%	2 5.9%	1 2.9%		33 38.4%	41 47.7%	9 10.5%	3 3.5%	
AE severity					0.004					<0.001
Moderate	35 15.4%	143 63.0%	37 16.3%	12 5.3%		2 6.8%	10 33.3%	13 43.3%	5 16.7%	
Severe	20 31.8%	26 41.3%	10 15.9%	7 11.1%		44 43.6%	49 48.5%	3 3.0%	5 4.6%	
PEPFAR AE type					<0.001					<0.001
Other	54 66.7%	21 25.9%	4 4.9%	2 2.5%		0 0.0%	9 64.3%	4 28.6%	1 7.1%	
Infection	1 0.5%	148 70.8%	43 20.6%	17 8.1%		1 4.4%	4 17.4%	9 39.1%	9 39.1%	
Device displacement	-	-	-	-		45 47.9%	46 48.9%	3 3.2%	0 0.0%	

*Results from chi-square tests.

**% are for within-category row proportions.

Among PrePex AEs, AE severity significantly varied over time (p<0.001) with the vast majority of severe AEs due to device displacements that, by definition, were all categorized as severe. AE type also differed significantly over time (p<0.001). As expected, device displacements, the most common PrePex AE, occurred before scheduled removal at Day 7. Infections were diagnosed later, accounting for most AEs identified on or after Day 15. Timing of AEs did not differ by age group (p<0.06).

## Discussion

This cross-sectional review of clients with AEs found that surgical and PrePex AEs were most common before routine Visit 2. On average, AEs occurred 7.5 and 5.6 days after the procedure or placement for surgical and PrePex clients, respectively. The most common early AE detected at or before Visit 1 was bleeding for surgical clients and device displacement for PrePex clients. For PrePex clients, nearly 90% (117/131) of all PrePex AEs were either device displacements or infections. Infection was most common among AEs occurring after Visit 1 for both methods. Although AEs were more common before Visit 2, almost 23% and 8% of surgical and PrePex AEs, respectively, occurred after this scheduled visit.

As would be expected, infections were most the common AE in the period after Visit 2 for both MC methods. Among those with more detailed reports, poor hygiene, dirty underwear, and lack of wound care appear common factors for those with detected infections, issues considered previously [19]. Among those with infections detected after Visit 2, some men reportedly attended both Visit 1 and 2, suggesting that these AEs developed after these earlier reviews. It is possible that others, still, had AEs after Visit 2 but did not seek care or sought care outside of ZAZIC-supported sites. As the schedule is implemented and disseminated, it is possible that the current visit schedule may give men a false sense of security that if they were healing well on Visits 1 and 2, they should have no further concerns. Improved post-operative counseling [20], including reinforcing the potential for complications at any time and education on warning signs of poor healing [21], could help improve clients’ prompt care seeking behaviors until fully healed [19].

Although younger boys ages 10–14 represent the majority of program MCs, they are not more likely, overall, to have an AE than their older peers [15]. However, younger boys are 3 times more likely to have infections than older peers, aged 20 and above [15]. In this current study among clients with AEs, boys ages 10–14 comprised 62% (179/290) of all surgical AEs and 68% (143/209) of all surgical infection AEs. In post hoc analysis among surgical clients who had an AE, young clients were more likely to have a severe AE than their older peers: 49% (31/63) of all severe surgical AEs occurred among boys ages 10–14 (p = 0.019). Findings from this analysis also suggest that, among clients who experienced an AE, later AEs were more common among younger clients and these later AEs were more likely to be infections.

Despite MC program age skew, and the differences in AE characteristics by age, routine MC counseling and care messages remain similar across all age groups. Younger MC patients warrant targeted attention to prevent, promptly identify, and manage AEs. A previous, multi-country study on adolescent wound care knowledge and practices found that adolescent MC clients reported multiple obstacles to proper post-operative wound care, including that care givers often lacked the correct information to help them [22]. Providers also noted concerns that younger patients, especially those ages 10–14, may be less able to understand post-MC instructions or independently adhere to proper wound care [23], potentially putting them at increased risk of an AE. Alternatively, younger men may not identify AEs early or may not report abnormal healing to caregivers early enough, possibly increasing the severity of AEs among this youngest age group. Within the MC counseling context, previous research found that young adolescents showed gaps in correctly recalling HIV-related content [24, 25]. Therefore, improved, age-appropriate, post-operative counseling would likely help ensure that younger clients comprehend and recall correct information regarding MC procedures and post-operative care. Simple educational materials provided to caregivers and guardians during the consent or counseling sessions or wider materials provided to communities could also help these younger boys receive the care they need at home to heal well [26]. Although some parents may encounter resistance from older adolescents in supervising post-operative care [27], engaging guardians in the need to be vigilant the first 2 or 3 weeks, including daily observation when possible, could encourage prompt care-seeking for any complication. Moreover, optimal post-operative counseling advises a clean care approach, including wearing clean underwear during healing [28]; however, young or poor clients are unlikely to purchase these garments independently. Consideration of supplying tighter underwear for the first days of healing to protect the wound and elevate the penis as well as providing clean, looser-fitting underwear to allow for subsequent healing over the recovery period could also reduce infection.

This study fills gaps in the literature. Although many previous studies report on moderate and severe AEs, noting rates ranging from 0.5% to 8% [14, 19, 29–40], these data come from controlled trials and pilot programs with active surveillance, conditions that are largely dissimilar to the routine program implementation setting discussed here. To the authors’ knowledge, few publications detail information on AEs within MC programs operating at scale or within routine program settings. Reed et al [19] examined follow-up rates for men who attended follow-up visits and those presumed lost to follow-up; however, they looked at clients only through Day 14, potentially missing AEs that came after that date. A study comparing AEs identified from active to those reported via passive surveillance systems in Kenya did not focus on the timing of AEs, specifically, but did note the average time of AE detection at 9 days post MC [12]. Lastly, in South Africa, a prospective study including post-surgical MC reviews at days 2,7 and 21 reported mean time to AE of 7 days, finding adherence to follow-up declined from 76% on Day 2 to 26% on Day 21[41]. Although highly informative, these special studies employed various levels of active surveillance, reducing the applicability of results for MC programs operating at scale. Therefore, the findings of this study fill a gap in the literature by discussing all AEs identified in the passive surveillance system employed in a routine program setting, potentially improving translation of results to inform MC policy and practice.

## Limitations

This study has several limitations. First, the sample size limited our ability to conduct additional types of analysis, including looking at differences between surgical procedures, forceps guided and dorsal slit. Additionally, clustering in AE timing due to routinely scheduled visits skewed the data distribution. Simpler nonparametric methods were used to prioritize results interpretation over more complicated regression models adjusted for AE timing and potential facility clustering. Due to limitations in data availability and feasibility for this quality assurance activity, we did not further examine AE rates by MC method or age group published previously [15]. Also, in alignment with updated PEPFAR policy, ZAZIC rolled out its AE Reporting Tool in summer, 2014, completing the form scale up in October, 2014. There is complete date on AEs that occurred between March 2013 and October 2014; only 25 are included from this period. We included all available AEs with detailed reports in this analysis to reflect programmatic realities, maximize data use, and increase the utility of results. It is possible that the excluded 196 AEs differed in some way; therefore, the findings of this analysis may better represent AEs from routine program implementation after October, 2014. Lastly, men could seek follow-up care for AEs outside of the ZAZIC program context in other private or public facilities, potentially leading to underreporting of ZAZIC AEs. Despite these limitations, we believe the results present informative findings from a large-scale MC program under routine implementation conditions.

## Conclusions

These findings suggest several programmatic steps that may improve quality service provision. First, it is important to remind clinicians that the period between Visit 2 and Visit 3 remains a period of high vigilance. Providers should be aware of this risk period when counseling or reviewing patients, reinforcing the message that men may seek care at any time if complications arise, before or after scheduled visits. Second, it is critical that routine counseling better alert clients that AEs can occur after routine scheduled visits to ensure patient vigilance to potential AEs. Better counseling on Visit 2 would further remind men to pay attention to their continued healing process and return immediately for any concerns. Third, as MC coverage aims to reach 90% of men ages 10–29 by 2021 in Zimbabwe [42], precautions should be taken to ensure safety for the 10–14 year old age group. For these youngest clients, especially, age-appropriate MC counseling and post-operative care materials are needed. These materials should be shared with, or tailored for, caregivers to help improve wound care and, ultimately, reduce complications. Finally, additional operational research on predictors of AEs in Zimbabwe or the region is warranted to help further improve patient safety. While global AE rates are low, absolute numbers of global AEs are likely in the hundreds of thousands. Utilizing the central collection of AE data by PEPFAR for further analyses might offer important opportunities for improving patient care.

## Supporting information

S1 Text Study dataset. (TXT)
