# Correction: Timing of adverse events among voluntary medical male circumcision clients: Implications from routine service delivery in Zimbabwe

**DOI:** 10.1371/journal.pone.0205113

**Published:** 2018-09-27

**Authors:** Caryl Feldacker, Aaron F. Bochner, Vernon Murenje, Batsirai Makunike-Chikwinya, Marrianne Holec, Sinokuthemba Xaba, Shirish Balachandra, John Mandisarisa, Vuyelwa Sidile-Chitimbire, Scott Barnhart, Mufuta Tshimanga

## Notice of republication

An incorrect version of S1 Text was published in error. This article was republished on September 19, 2018 to correct for this error. Please download this article again to view the correct version.
